# Optical coherence tomography angiographic findings of lamellar macular hole: comparisons between tractional and degenerative subtypes

**DOI:** 10.1038/s41598-020-70254-0

**Published:** 2020-08-07

**Authors:** Joon Hyung Yeo, Richul Oh, Joo Yong Lee, June-Gone Kim, Young Hee Yoon, Yoon Jeon Kim

**Affiliations:** 1 Department of Ophthalmology, Chung-Ang University Hospital, Chung-Ang University College of Medicine, Seoul, Republic of Korea; 2 Department of Ophthalmology, Seoul National University College of Medicine, Seoul, Republic of Korea; 3 Department of Ophthalmology, Asan Medical Center, University of Ulsan College of Medicine, 88, Olympic-ro 43-gil, Songpa-gu, Seoul, 05505 Republic of Korea

**Keywords:** Eye diseases, Eye manifestations, Imaging and sensing

## Abstract

We investigated the microvascular changes in eyes with lamellar macular holes (LMHs) using optical coherence tomography angiography (OCTA), compare them between two subtypes of LMH. Tractional and degenerative LMH were differentiated based on the morphological characteristics of OCT. In OCTA images, foveal and parafoveal vessel density (VD) in the superficial and deep capillary plexus (SCP, DCP) and foveal avascular zone (FAZ) area were measured. Eyes that underwent vitrectomy for LMH were included in subgroup analysis. We analysed 63 LMH (42 tractional and 21 degenerative) eyes and 63 control eyes. Compared with degenerative LMH, tractional LMH had better BCVA (*p* = 0.010), smaller FAZ area (*p* = 0.001), and higher foveal VD in the SCP (*p* = 0.130) and DCP (*p* = 0.002). In degenerative LMH, better BCVA was associated with greater foveal VD in the SCP (*p* = 0.040) and DCP (*p* = 0.005), and parafoveal VD in the SCP (*p* = 0.006). In subgroup analysis, only the tractional LMH group showed significant increases in foveal and parafoveal VDs in the SCP after vitrectomy (*p* = 0.001 and *p* = 0.026, respectively). Significant differences in microvascular changes were noted between tractional and degenerative LMH, suggesting that two subtypes are distinct pathogenetic entities.

## Introduction

Lamellar macular hole (LMH) is a vitreoretinal disorder characterised by partial-thickness foveal defect with irregular foveal contour and dehiscence of the inner foveal retina from the outer retina^1^. LMH was first described by Gass in 1975^2^; however, its pathophysiologic mechanisms remain poorly understood^3^. In addition, a consensus among clinicians over the progression of LMH has yet to be reached. A small proportion of patients with LMH develop severe visual impairment and/or show anatomical signs of progression on optical coherence tomography (OCT)^4–6^.

With the advent of multimodal imaging in the evaluation of retinal disorders, many authors have studied the morphological and functional characteristics of LMH, which could help distinguish between different pathological forms^4,7,8^. Recently, Govetto et al. proposed that LMH consists of two subtypes—tractional and degenerative—based on the structural differences observed on OCT imaging^9^. While tractional LMH is characterized by a sharp-edged, “schisis-like” appearance and tractional epiretinal membrane (ERM), degenerative LMH has round-edged cavitation, a foveal bump, and is often associated with lamellar hole-associated epiretinal proliferation. It was also reported that visual acuity is worse in degenerative LMH than in tractional LMH^4,9^.

The pathogenesis of LMH is yet to be fully investigated. The recent advent of OCT angiography (OCTA) has enabled the visualization of the retinal vascular network. Based on the concept of neurovascular units in the retina, the pattern and extent of neurodegenerative changes can be inferred through vascular changes. However, no studies have yet to use OCTA to compare the vascular changes in the two subtypes of LMH. We herein investigated the microvascular changes in patients with LMH using OCTA, compared them between the two subtypes of LMH, and determined their associations with visual function.

## Results

A total of 63 eyes of 63 patients with LMH (17 men, 46 women) and 63 eyes of 63 normally sighted individuals were included in this study. The mean age of the study population was 65.4 ± 6.4 years (range 48–86). Of the study eyes, 42 eyes were diagnosed with tractional LMH, and the other 21 eyes were diagnosed with degenerative LMH. The demographic and clinical characteristics of the study patients and control subjects are summarized in Table 1. Compared with control eyes, eyes with LMH had significantly worse BCVA (0.22 ± 0.17 logMAR, *p* < 0.001). Between the two subtypes LMH, there were no significant differences regarding age, refractive errors, axial length, and proportion of pseudophakic eyes (Table 1); however, BCVA was worse in the degenerative LMH group (0.30 ± 0.18 logMAR) than in the tractional LMH group (0.18 ± 0.16 logMAR, *p* = 0.010).

**Table 1:** Demographic and clinical characteristics of patient and control eyes.

	Tractional LMH (n = 42)	Degenerative LMH (n = 21)	Controls (n = 63)	*p* values
Mean age, years	65.3 (7.3)	66.0 (6.5)	65.4 (6.5)	0.980*
Range, years	48–78	53–86	48–86	
Female, n (%)	36 (85.7)	10 (47.6)	39 (61.9)	0.004^†^
BCVA, logMAR	0.18 (0.16)	0.30 (0.18)	0.04 (0.07)	< 0.001*
Refractive errors, D	− 0.27 (2.15)	− 0.56 (1.86)	− 0.06 (1.57)	0.416 *
Pseudophakia, n (%)	4 (9.5)	5 (23.8)	13 (20.6)	0.239^†^
Axial length, mm	23.5 (1.0)	23.8 (1.4)	23.7 (1.3)	0.910*

*BCVA* best-corrected visual acuity, *LMH* lamellar macular hole, *logMAR* logarithm of the minimum angle of resolution.

Data are presented as mean (standard deviation) unless indicated otherwise.

* Kruskal–Wallis test.

^†^Chi-squared test.

The morphologic differences observed in OCT between the two groups are shown in Table 2. The tractional LMH group had significantly higher central foveal thickness, parafoveal thickness, and minimum foveal retinal thickness (*p* = 0.007, *p* = 0.048, and *p* < 0.001, respectively), while the degenerative LMH group had a larger inner diameter and a smaller outer diameter than the tractional LMH group (*p* = 0.021 and *p* < 0.001).

**Table 2:** Optical coherence tomography parameters of the eyes with lamellar macular hole and control eyes.

	Tractional LMH (n = 42)	Degenerative LMH (n = 21)	*p* values	Controls (n = 63)	*p* values**
ERM, n (%)	41 (97.62)	20 (95.24)	0.988*	–	–
LHEP, n (%)	3 (7.14)	13 (61.90)	< 0.001^†^	–	–
EZ disruption, n (%)	2 (4.76)	15 (71.43)	< 0.001^†^	–	–
Central foveal thickness, µm	413.17 (64.48)	363.38 (70.75)	0.007^‡^	246.87 (20.24)	< 0.001
Parafoveal thickness, µm	393.09 (39.56)	371.41 (41.55)	0.048^‡^	319.00 (17.34)	< 0.001
Minimum foveal retinal thickness, µm	185.49 (25.95)	150.38 (32.60)	< 0.001^‡^	204.05 (17.61)	< 0.001
Inner diameter, µm	483.00 (171.95)	590.29 (165.65)	0.021^‡^	–	–
Outer diameter, µm	1,530.02 (621.26)	722.86 (270.46)	< 0.001^‡^	–	–

*ERM* epiretinal membrane, *LHEP* lamellar hole-associated epiretinal proliferation, *EZ* ellipsoid zone, *LMH* lamellar macular hole.

Data are presented as mean (standard deviation) unless indicated otherwise.

*Statistical comparison between tractional LMH and degenerative LMH using Fisher’s exact test.

^†^Statistical comparison between tractional LMH and degenerative LMH using the Chi-squared test.

^‡^Statistical comparison between tractional LMH and degenerative LMH using Student’s *t*-test.

**Statistical comparison among tractional LMH, degenerative LMH, and controls using the Kruskal–Wallis test.

**Figure 1:**
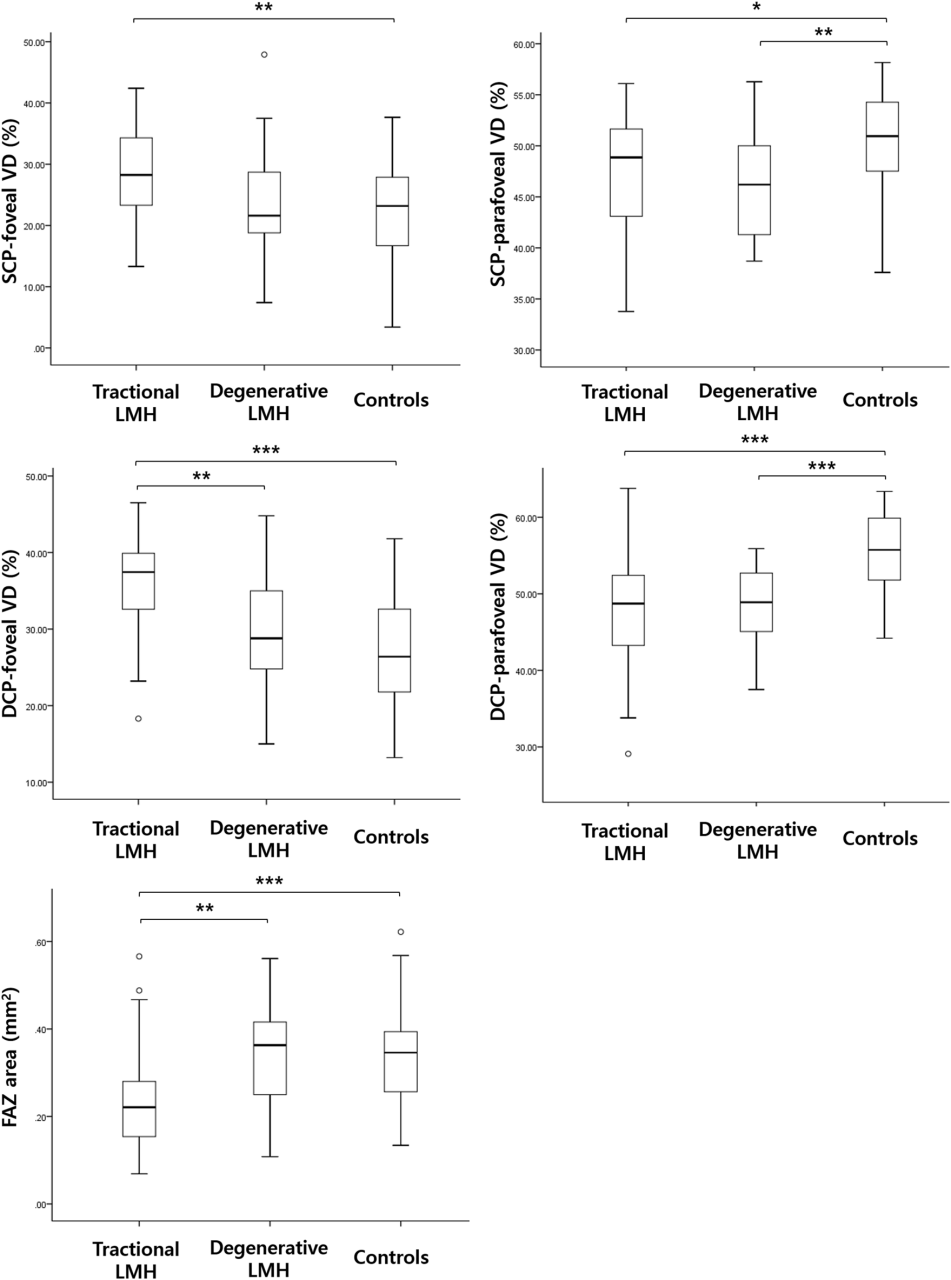
Vessel densities and foveal avascular zone areas in eyes with tractional lamellar macular hole (LMH), degenerative LMH, and control eyes. *p < 0.05, ** *p* < 0.01, *** *p* < 0.001. *DCP* deep capillary plexus, *FAZ* foveal avascular zone, *LMH* lamellar macular hole, *SCP* superficial capillary plexus, *VD* vessel density.

Table 3 summarizes the macular microvascular characteristics of the LMH and control groups. The foveal vessel density (VD) and parafoveal VD were significantly different among the three subgroups, both at the superficial capillary plexus (SCP) and the deep capillary plexus (DCP). In the tractional LMH group, the foveal VDs measured at the SCP and DCP were significantly higher than those of the control group. The parafoveal VDs were significantly lower than those of the control group (Fig. 1). In addition, the vessel diameter index (VDI) measured at the SCP and DCP was significantly lower than that of the control group. In the degenerative LMH group, the parafoveal VDs measured at the SCP and DCP were significantly lower than those of the control group, whereas no significant difference was noted in the foveal VD (Fig. 1). A statistically significant difference was found between degenerative LMH and controls for VDI measured at the SCP.

**Table 3:** Optical coherence tomography angiography parameters of the eyes with lamellar macular hole and control eyes.

	Tractional LMH (n = 42)	Degenerative LMH (n = 21)	Controls (n = 63)	*p* values*	*p* values^†^	*p* values^‡^
SCP						
Foveal VD, %	28.40 (7.54)	23.80 (9.76)	22.16 (7.97)	0.130	0.001	0.979
Parafoveal VD, %	47.08 (5.77)	46.14 (5.11)	50.50 (4.71)	0.910	0.017	0.003
VLD	0.12 (0.02)	0.11 (0.02)	0.12 (0.01)	0.301	0.278	0.053
VDI	2.21 (0.09)	2.25 (0.14)	2.31 (0.07)	0.377	0.001	0.004
DCP						
Foveal VD, %	36.33 (6.13)	29.34 (8.24)	26.85 (7.50)	0.002	< 0.001	0.510
Parafoveal VD, %	47.83 (7.01)	48.58 (5.27)	55.62 (4.88)	0.998	< 0.001	< 0.001
VLD	0.17 (0.02)	0.17 (0.02)	0.18 (0.01)	0.445	0.200	0.275
VDI	1.82 (0.04)	1.83 (0.07)	1.85 (0.04)	0.520	0.002	0.342
FAZ, mm^2^	0.23 (0.11)	0.35 (0.13)	0.33 (0.10)	0.001	< 0.001	0.998

*LMH* lamellar macular hole, *SCP* superficial capillary plexus, *VD* vessel density, *VLD* vessel length density, *VDI* vessel diameter index, *DCP* deep capillary plexus, *FAZ* foveal avascular zone.

Data are presented as mean (standard deviation) unless indicated otherwise. Statistical analysis was performed using the Kruskal–Wallis test with Bonferroni correction.

*Statistical comparison between tractional LMH and degenerative LMH.

^†^Statistical comparison between tractional LMH and controls.

^‡^Statistical comparison between degenerative LMH and controls.

The foveal avascular zone (FAZ) area was also different among groups (*p* < 0.001). Post-hoc analysis showed that tractional LMH had a smaller FAZ than did the degenerative LMH group and the control group (Fig. 1).

**Figure 2:**
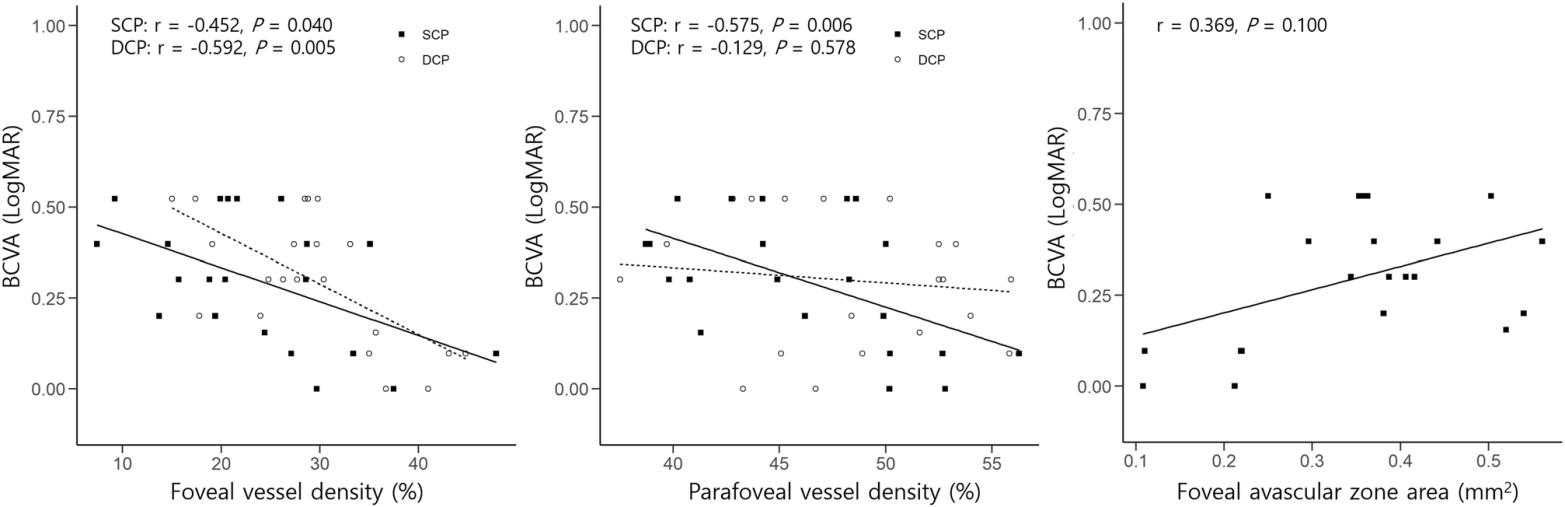
Scatter plots showing the correlations between the best-corrected visual acuity (BCVA) and the foveal and parafoveal vessel density in both plexuses or fovea avascular zone area in eyes with degenerative lamellar macular hole. Spearman’s correlation coefficient (r) and *p* values for the slope of the regression line are noted. *DCP* deep capillary plexus, *SCP* superficial capillary plexus.

We also investigated the associations of BCVA with ocular structural factors and OCTA parameters. None of the structural parameters regarding diameter and thickness were significantly correlated with BCVA in both LMH subtypes. Among the OCTA parameters, foveal VDs in both plexuses and parafoveal VD in SCP were negatively correlated with BCVA in degenerative LMH, indicating that eyes with degenerative LMH with smaller VD had worse BCVA (Fig. 2). In contrast, no significant correlations were identified between the OCTA parameter and BCVA in the tractional LMH group.

Of the 63 study eyes, 49 eyes underwent vitrectomy for LMH, 24 of which had available OCTA images (15 eyes in tractional LMH and 9 eyes in degenerative LMH) and included in subgroup analysis. Table 4 shows the OCTA parameters before and after vitrectomy. Foveal/parafoveal VDs and VDI in SCP of the eyes with tractional LMH increased significantly after surgery (*p* = 0.001, *p* = 0.026, and *p* = 0.020, respectively). However, in eyes with degenerative LMH, no statistically significant changes were observed except in vessel length density (VLD) in DCP (*p* = 0.028).

**Table 4:** Comparisons between the preoperative and postoperative optical coherence tomography angiography parameters of the eyes with tractional and degenerative lamellar macular hole.

	Tractional LMH (n = 15)			*p* values*	Degenerative LMH (n = 9)		*p* values*
	Preop	Postop			Preop	Postop	
SCP							
Foveal VD, %	25.51 (6.98)	31.25 (5.64)		0.001	25.63 (11.15)	27.47 (9.03)	0.496
Parafoveal VD, %	44.20 (6.63)	47.96 (3.56)		0.026	46.07 (5.92)	44.71 (6.04)	0.496
VLD	0.11 (0.02)	0.10 (0.02)		0.251	0.11 (0.03)	0.10 (0.02)	0.515
VDI	2.21 (0.10)	2.31 (0.17)		0.020	2.27 (0.18)	2.31 (0.13)	0.374
DCP							
Foveal VD, %	36.94 (4.16)	36.95 (5.56)		0.720	29.11 (10.14)	35.18 (9.15)	0.055
Parafoveal VD, %	48.44 (7.13)	51.89 (3.35)		0.095	48.18 (6.13)	52.41 (5.62)	0.098
VLD	0.18 (0.02)	0.16 (0.02)		0.120	0.18 (0.03)	0.15 (0.03)	0.028
VDI	1.83 (0.04)	1.82 (0.04)		0.560	1.86 (0.07)	1.84 (0.07)	0.441
FAZ, mm^2^	0.23 (0.07)	0.22 (0.09)		0.600	0.33 (0.12)	0.25 (0.13)	0.039

*LMH* lamellar macular hole, *Preop.* Preoperative, *Postop.* Postoperative, *SCP* superficial capillary plexus, *VD* vessel density, *VLD* vessel length density, *VDI* vessel diameter index, *DCP* deep capillary plexus, *FAZ* foveal avascular zone.

Data are presented as mean (standard deviation) unless indicated otherwise.

*Wilcoxon signed-rank test.

## Discussion

Vitreoretinal disorders such as full-thickness macular hole, LMH, and macular pseudohole alter the retinal vascular networks in distinct ways^10,11^, and these changes may offer new perspectives on the pathophysiology of vitreoretinal disorders. To the best of our knowledge, microvascular changes occurring in different subtypes of LMH have yet to be reported. In this study, we assessed the retinal microvascular changes using OCTA and compared them between eyes with tractional LMH and those with degenerative LMH. Compared with control eyes, those with tractional LMH had smaller FAZ area, higher foveal VD, lower parafoveal VD, and lower VDI in both plexuses. Eyes with degenerative LMH had lower parafoveal VD in both plexuses and lower VDI in SCP than did the control eyes. In addition, foveal VDs in both plexuses and parafoveal VD in SCP were significantly correlated with BCVA in eyes with degenerative LMH.

On the basis of our findings in eyes with tractional LMH, we can infer that tractional LMH shares similar pathogenic pathways with ERM. ERM contracture results in tangential traction on the underlying inner and outer retinal layers^12–15^. Previous studies using OCTA revealed retinal microvascular alterations secondary to ERM as well as retinal morphological changes, which are similar to our results^15–18^. In addition, patients with ERM showed a significantly slower blood flow velocity in perifoveal capillaries than did healthy individuals^19,20^; such reduced blood velocity was restored after surgical removal of ERM, suggesting that tractional forces of the ERM may increase the venous resistance and reduce the capillary blood flow^19,21,22^. Interestingly, our results showed that foveal and parafoveal VDs in the SCP of eyes with tractional LMH had increased significantly at 6 months postoperatively, whereas eyes with degenerative LMH did not show such changes; this might be due to the restoration of microvascular alteration with releasing of the tractive force by ERM. In addition, preoperative reduction and postoperative restoration of vessel diameter, described by Mastropasqua et al. in ERM patients^18^, were also observed in the tractional LMH group of our study. In this context, it is plausible that ERM and tractional LMH represent two sides of the same spectrum of disease, which is in line with the results of a recent study that proposed an OCT- based definition of LMH to better distinguish LMH from similar conditions^23^. It is speculated that a linear and continuous ERM over the entire macula area can exert centripetal traction on the fovea, resulting in a typical appearance of ERM. If the foci of the retinal contraction in ERM are located in the extrafoveal area, it may result in centrifugal traction of ERM, contributing to the development of tractional LMH.

On the other hand, despite the clinical similarity between tractional LMH and degenerative LMH, our results showed significant differences in terms of vascular changes between the two subtypes. Although ERM was present in most eyes with degenerative LMH, there were no significant differences in the foveal VDs in both plexuses and FAZ area between the degenerative LMH group and the control group. Notably, unlike eyes with tractional LMH, those with degenerative LMH did not show significant postoperative improvement in parafoveal VD. Moreover, eyes with degenerative LMH did not show statistically significant differences in postoperative VDI measured at the SCP, although a trend towards an increase compared to preoperative values was observed. This difference suggests that the traction forces exerted by ERM in degenerative LMH may be different from those in tractional LMH, and that ERM may not play a leading role in the pathophysiology of degenerative LMH.

The present study further demonstrated that compared with control eyes, those with degenerative LMH showed lower parafoveal VD in the SCP and DCP and a frequent defect of the ellipsoid zone. In addition, BCVA in the degenerative LMH group was significantly correlated with the microvascular alterations; specifically, smaller foveal VDs in both plexuses and smaller parafoveal VD in SCP were correlated with worse BCVA. Microvascular reduction may induce visual impairment via the disruption of the photoreceptors. Because 10–15% of oxygen supplementation to photoreceptors are derived from the DCP, relative hypoperfusion in the DCP may decrease the integrity of photoreceptors, resulting in the worse BCVA in this group^24–26^. The association between visual acuity and vascular changes, however, were not identified in eyes with tractional LMH. Taken together, we suggest that the pathophysiology of degenerative LMH represents a pathway distinct from that of tractional LMH, in which traction of ERM plays a pivotal role. Microvascular alterations in degenerative LMH, which was characterized by lower parafoveal VD, may reflect a slow, chronic, degenerative process that causes the loss of retinal tissue and the disruption of the ellipsoid zone.

As LMH is considered to be relatively stable and only rarely progresses to more severe levels of visual deficit^3–6^, the optimal timing for surgical intervention in LMH remains controversial. Moreover, Govetto et al. reported that the BCVA remained stable in both tractional and degenerative LMH^9^. Our results revealed no significant correlations between ocular structural factors and BCVA in both subtypes of LMH, which means visual function remains stable despite some degree of anatomic progression, thus supporting the idea that observation without surgical removal might be a tolerable option. Although a relatively small number of eyes with degenerative LMH were included in this study, BCVA was found to be significantly correlated with changes in VD. Therefore, we presume that microvascular alterations, but not ocular structural factors, may be a biomarker of visual prognosis in patients with degenerative LMH.

The present study has several limitations. First, the sample size was relatively small, and 9 eyes with degenerative LMH were included in the vitrectomy subgroup analysis. As a result, we were unable to investigate the correlation between preoperative ocular factors and postoperative visual outcomes. Nevertheless, our study holds value because this is the first to investigate the microvascular changes according to different subtypes of LMH using OCTA. Second, confounding factors such as blood pressure may have interfered with our study results. Ideally, the study should have enrolled patients with unilateral LMH and compared these eyes with healthy fellow eyes; however, as the number of patients with unilateral LMH was too small to draw meaningful conclusions, we also included patients with bilateral LMH and normally sighted age-matched controls. Third, due to the cross-sectional design of the study, we were unable to properly assess the cause-and-effect relationship between microvascular changes and the development or progression of LMH. Finally, we only used BCVA as the index for visual function. Considering that retinal sensitivity or metamorphopsia affects the visual function, assessment of various aspects of visual outcomes is needed.

In conclusion, we observed significant differences in the microvascular alterations between tractional and degenerative LMHs using OCTA and found that microvascular impairments were significantly correlated with visual outcomes in eyes with degenerative LMH. The changes noted in eyes with tractional LMH were similar to those commonly observed in ERM. These results support the idea that tractional and degenerative LMH are distinct pathogenic entities. A future study using serial observation of retinal vascular changes throughout follow-up will further add to our understanding of the pathophysiology of LMH.

## Methods

All procedures were conducted in accordance with the tenets of the Declaration of Helsinki, and the study design was approved by the Institutional Review Board (IRB) of Asan Medical Center (Seoul, Korea; IRB No. 2019-0131). Due to the retrospective design of the study and the use of de-identified patient data, the IRB of Asan Medical Center waived the need for written informed consent from patients and control subjects.

### Patients

A retrospective consecutive chart review was conducted for all patients diagnosed with LMH and underwent OCTA during follow-up at the retina clinic of Asan Medical Center between December 2016 and November 2019. LMH was defined as an irregular foveal contour, break at the inner fovea, or intraretinal splitting, as previously described^1^. All OCT images were carefully reviewed by two investigators (J.H.Y. and Y.J.K.) in a blinded fashion. Determination of LMH subtypes was performed according to the previously established OCT criteria including the presence of lamellar hole-associated epiretinal proliferation, presence of typical ERM, presence of foveal bump, integrity of the ellipsoid layer, shape of intraretinal separation (round-edged cavitation or sharp-edged split), and the ratio between inner and outer diameter^9^. The subtype of LMH was determined by a consensus between two investigators.

Exclusion criteria for the study eye included secondary ERM (e.g., retinal detachment, diabetic retinopathy, retinal vascular occlusions, uveitis, trauma), exudative age-related macular degeneration, glaucoma, media opacity with insufficient OCTA image quality, and history of vitrectomy. In addition, patients with a history of vitrectomy or refractive error (spherical equivalent) of − 6 diopters or greater were excluded. For control, we included normally sighted age-matched subjects without ocular diseases, history of ocular surgery, or systemic diseases that have known effects on retinal vasculatures such as diabetes or hypertension.

Demographic and ophthalmological data collected from patient records included age, sex, best-corrected visual acuity (BCVA), refractive error, lens status, and axial length. BCVA in Snellen values was converted to the logarithm of the minimum angle of resolution for statistical analysis. Eyes that underwent vitrectomy for LMH during follow-up and had postoperative data including BCVA and OCTA images were included in the subgroup analysis.

### Optical coherence tomography

**Figure 3:**
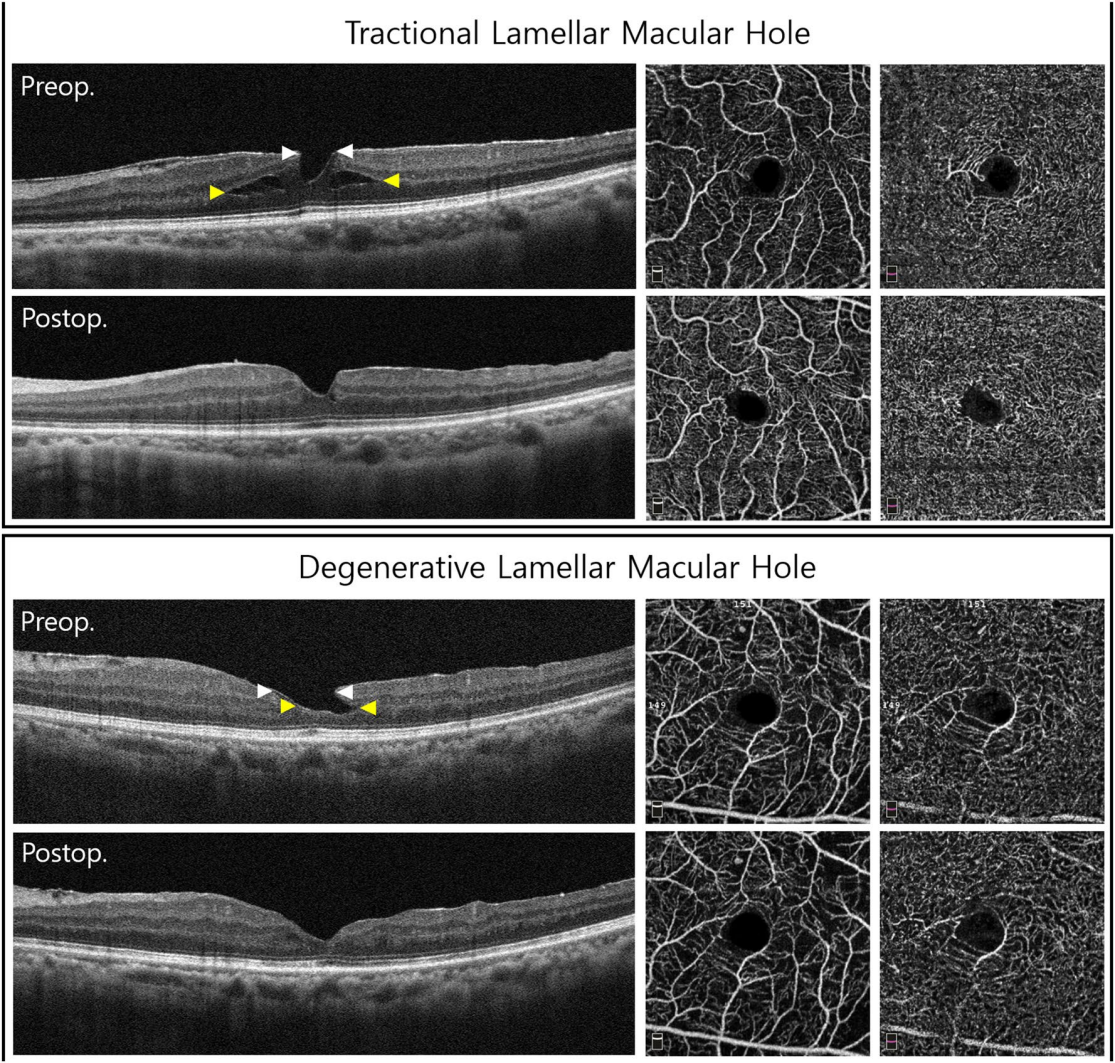
Preoperative and postoperative optical coherence tomography (OCT) and OCT angiography of the eyes with tractional and degenerative lamellar macular hole (LMH). (Top) Tractional LMH. The inner diameter (white arrowheads) measured at the level of the internal limiting membrane was 345 µm. The outer diameter (yellow arrowheads), the maximum diameter of the intraretinal schisis, was 1,670 µm. After the tractional epiretinal membrane was removed in surgery, the foveal and parafoveal vessel densities in the superficial capillary plexus increased. In addition, foveal avascular zone (FAZ) area increased from 0.191 to 0.214 mm^2^. (Bottom) Degenerative LMH. Compared with tractional LMH, degenerative LMH had a larger inner diameter (400 µm, white arrowheads) and a smaller outer diameter (506 µm, yellow arrowheads). No significant microvascular changes were observed at 6 months after surgery. Postoperative FAZ area was 0.249 mm^2^, with no significant difference from the preoperative FAZ area (0.250 mm^2^). *Preop.* Preoperative, *Postop.* postoperative.

Optovue RTVue XR AVANTI version 2018.1.0.37 (Optovue, Fremont, CA, USA) was used to obtain all standard cross-sectional scans. We selected the 6-mm retinal map analysis protocol, with 250-µm intervals in the central 4 mm, for the volume scans to reconstruct a surface map with numeric averages of the measurements for each of the 9 map sectors as defined by the Early Treatment Diabetic Retinopathy Study (ETDRS). The tomographic parameters obtained from OCT images were the following; presence of ERM, presence of LHEP, integrity of the ellipsoid zone (intact/disruption), minimum foveal retinal thickness, mean central foveal/parafoveal thickness, maximum diameter of the edge of the hole at the level of the retinal surface (inner diameter), and the maximum intraretinal diameter (outer diameter, Fig. 3). Diameters and minimum foveal retinal thickness were independent measured by two investigators (J.H.Y. and R.O.) based on the OCT B-scan images as previously described^9^. Each investigator took five measurements from one image and discarded the maximal and minimal value, and the mean value of the remaining six measurements was used in subsequent analyses.

### Optical coherence tomography angiography

Optovue RTVue XR AVANTI with AngioVue was used to acquire OCTA images. OCTA was acquired in the 3 × 3 mm^2^ macular area. Each full-thickness retinal scan was segmented as follows: the SCP from the inner limiting membrane (ILM) to the outer limits of the inner plexiform layer (IPL), and the DCP from the outer limits of the IPL to the outer limits of the outer plexiform layer. The tomographic parameters obtained from OCTA images were the following; foveal/parafoveal VD in the SCP and DCP, FAZ area, VLD, and VDI. VD was calculated as the percentage of the area occupied by vessels in the selected region. The foveal VD and parafoveal VD were defined as the vessel density in the foveal region with a diameter of 1 mm and that in the parafoveal region with a diameter of 1–3 mm, respectively, and separately calculated in five regions (i.e., fovea, tempo, superior, nasal, and inferior) based on the ETDRS grid sectors. The average values of four parafoveal sectors were used for analyses. The FAZ area was manually outlined through the freehand selection tool, and its dimensions were calculated using the instrument’s internal software.

A custom semi-automated algorithm was developed and used to quantify several parameters (VLD and VDI). To quantify these parameters, the grayscale 2D en face OCTA images segmented at the SCP and DCP level were imported into ImageJ 1.50 software (National Institutes of Health, Bethesda, MD, USA) and converted into an 8-bit image. This image was converted into a binary image using fixed cutoff thresholding^27^. After acquiring the binary image, perfusion density was calculated as a unitless proportion of the number of pixels over the threshold divided by the total number of pixels in the analysed area. Skeletonized images were created by iteratively deleting the pixels in the outer boundary of the binarised, white-pixelated vasculature until 1 pixel remained along the width direction of the vessels. Based on the skeletonised image, the VLD was calculated as the percentage of area occupied by the vessel in the designated 3 × 3 mm^2^ area. The VDI was then calculated by dividing the value of perfusion density by the VLD, which yields the average vessel calibre.

All scans were independently reviewed by two investigators (J.H.Y. and R.O.) who were blinded to all clinical information to ensure correct segmentation and sufficient quality. In cases of incorrect segmentation, manual adjustment of the segmentation was performed using the AngioVue module of the Optovue RTVue XR AVANTI software installed in the instrument. We defined images of sufficient quality as those centred on the macula and without significant motion artefacts or edge duplication. Cases with insufficient image qualities were excluded from the study.

### Surgical technique

Twenty-four patients underwent 25-gauge pars plana vitrectomy with ERM and ILM peeling under local or general anaesthesia by one of the four surgeons with the Alcon Constellation vision system (Alcon Laboratories, Fort Worth, Texas, USA). Cataract surgeries were performed at the discretion of the surgeon. After performing a core vitrectomy, posterior vitreous detachment was induced using a vitreous cutter or extensible intraocular pick. In some cases, to ensure complete separation of the vitreous cortex from the retina, 20% diluted triamcinolone acetonide was injected into the vitreous cavity. After staining the posterior pole with 0.25% indocyanine green to enhance visualisation during ILM peeling, ILM forceps were used to peel the ERM or LHEP and the ILM up to the vascular arcades. At the end of the surgery, air–fluid exchange was performed at the surgeon’s discretion, eventually followed by air–gas exchange. An isoexpansile mixture of perfluoropropane gas (C_3_F_8_) was used. Postoperatively, these patients were requested to remain face down for 3–7 days.

### Statistical analysis

All statistical analyses were performed using IBM SPSS Statistics for Windows, version 20.0 (IBM Corp., Armonk, NY, USA). Continuous variables are presented as mean ± standard deviation. Intergroup comparisons for continuous variables were carried out by the Kruskal–Wallis test, Student’s *t*-test, or Wilcoxon signed-rank test as appropriate, and Bonferroni correction was for post-hoc tests. Intergroup comparisons for categorical variables were carried out by Chi-squared test or Fisher’s exact test as appropriate. Analysis of associations between BCVA and the data obtained from OCT/OCTA was performed using the Spearman’s correlation test. *p* values less than 0.05 were considered statistically significant.

## Data Availability

The datasets generated during and/or analyzed during the current study are available from the corresponding author on reasonable request.
